# *Astragalus* small molecules protect BMSCs from radiation-induced bystander effect and enhance lung cancer radiosensitivity via the primary cilium/TGF-βR1/Smad3 pathway

**DOI:** 10.3389/fonc.2026.1732029

**Published:** 2026-03-04

**Authors:** Zhiming Miao, Mengyuan Wu, Sichao Dai, Xin Wang, Yang Yang Li, Fuxian Liu, Zhiwei Liu, Liying Zhang, Yongqi Liu

**Affiliations:** 1 Gansu University of Chinese Medicine, Provincial Key Laboratory of Molecular Medicine and Prevention Research of Major Diseases, Lanzhou, China; 2 College of Integrated Traditional Chinese and Western Medicine, Jining Medical University, Jining, Shandong, China

**Keywords:** bystander effect, DNA damage, primary cilia, rosewood, TGFβR1, vanillic acid

## Abstract

**Background:**

Radiotherapy is an important treatment for lung cancer. However, in the course of radiotherapy, treatment-related side effects and decreased radiosensitivity remain challenging issues. TGF-βR1 can induce radiation-induced bystander effect (RIBE) through the primary cilia; however, this mechanism needs to be further elucidated. At present, traditional Chinese medicine (TCM) shows great advantages in protecting against RIBE, in which *Astragalus* and its related formulations show good protective effects against radiation; however, the mechanisms by which *Astragalus* exerts these protective effects are unknown. Therefore, this study aims to investigate the molecular mechanisms by which TGF-βR1 exerts RIBE through the primary cilia, enhancing radiosensitivity, and to reveal the therapeutic effects of small molecules derived from *Astragalus membranaceus* via this pathway.

**Methods:**

A co-culture model of A549 cells and bone marrow mesenchymal stem cells (BMSCs) was established, and network pharmacology was employed to identify key proteins involved in the repair of radiation-induced DNA damage in BMSCs. The role of the primary cilium/TGF-βR1 pathway in the repair of radiation-induced DNA damage in adjacent BMSCs was investigated using immunofluorescence and Western blot techniques. Molecular docking technology was utilized to screen effective small molecules from *Astragalus* that target the primary cilium/TGF-βR1 pathway. The screened effective small molecules were then combined, and their effects on radiation-induced bystander effect in neighboring BMSCs were studied through the CCK-8 assay, colony formation assay, apoptosis assay, cell cycle analysis, immunofluorescence, and Western blot experiments.

**Results:**

The core differentially expressed gene IFT88 was identified by bioinformatics analysis. In the co-culture model with BMSCs following A549 irradiation with 2 Gy of X-ray, BMSCs were inhibited. After irradiation, TGF-βR1, IFT88, and RAD51 were abnormally activated in the adjacent BMSCs. However, after knockdown of IFT88 (SiIFT88), the protein expressions of TGF-βR1 and RAD51 were significantly decreased. Based on molecular docking screening for TGF-βR1 and IFT88 using the *Astragalus* small molecule compounds vanillic acid and 3-hydroxy-9,10-dimethoxy rosewood, the expression of TGF-βR1 and RAD51 proteins and the number of primary cilia were decreased by the intervention of these two small molecules alone or in combination with radiation in paracellular and lung cancer cells, but the expression level of TGF-βR1 was not affected.

**Conclusion:**

Primary cilia play a key role in the repair of radiation-induced DNA damage in adjacent BMSCs and in enhancing the radiosensitivity of lung cancer. Vanillic acid and rosewood in *A. membranaceus* small molecules can regulate DNA damage in BMSCs through the TGF-βR1/primary cilia.

## 1 Introduction

Lung cancer is the most prevalent malignancy and the leading cause of tumor-related deaths worldwide (1). Non-small cell lung cancer (NSCLC) is the most common pathohistologic type of lung cancer, accounting for approximately 85% of all types. Current treatment modalities for NSCLC include surgery, chemotherapy, molecular targeted therapy, immunotherapy, and radiotherapy. Among them, radiotherapy is one of the most commonly used methods of lung cancer treatment, with more than 70% of lung cancer patients having indications for radiotherapy and more than 50% of patients receiving radiotherapy (2). Radiotherapy is the use of ionizing radiation to irradiate tumors, causing DNA double-strand breaks in tumor cells, thus inhibiting tumor growth (3). However, while radiation induces DNA damage in tumor cells and kills tumor cells, unirradiated normal cells and tissues are also induced to undergo biological effects similar to those of irradiated cells and tissues, causing inflammation, DNA damage, chromosomal aberrations, cell death, apoptosis, radiation adaptation, and other effects; this phenomenon is known as radiation-induced bystander effect (RIBE) (4, 5), which makes the radiotherapy of tumors more complicated, and even if it is a precise treatment, it still inevitably causes damage to normal tissues, which greatly reduces the benefit of radiotherapy. Moreover, during radiotherapy, the radiation sensitivity of cancer cells is easily reduced, resulting in a decrease in the effect of radiotherapy.

Bone marrow stromal cells (BMSCs) are pluripotent stem cells with self-renewal, multidirectional differentiation, and immunomodulatory functions, and they are one of the important mesenchymal components in the lung cancer microenvironment (6). It has been found that tumor cells can secrete a variety of chemokines to recruit BMSCs to gather around tumor cells and participate in the process of repairing tissue damage around the tumor (7, 8). However, due to the presence of RIBE, parabasal BMSCs are susceptible to damage when the organism undergoes radiotherapy for lung cancer, resulting in DNA damage and alteration of its own genetic stability, which ultimately leads to apoptosis or malignant transformation (9). Therefore, the search for the key molecular mechanisms of genomic damage caused by RIBE to BMSCs has attracted our extensive attention.

Currently, most mechanistic studies of RIBE have focused on cell gap junction communication, soluble factors secreted by target cells, and exosomes (10, 11). Several studies have demonstrated that soluble factors such as reactive oxygen species, nitric oxide, and transforming growth factor-β1 (TGF-β1) can mediate the development of RIBE. The transforming growth factor-beta (TGF-β) pathway is a critical mediator of RIBE. Upon activation, TGF-β1 binds to its type II receptor (TGF-βRII), which then recruits and phosphorylates the type I receptor (TGF-βRI), leading to the phosphorylation of downstream Smad2/3 proteins. This complex translocates to the nucleus to regulate gene expression, including those involved in DNA damage response (12, 13). Moreover, TGF-β1 is considered to be the most important cytokine to induce RIBE. Studies have found that radiation induced oncogenic transformation of surrounding normal tissues by high expression of TGF-β1 (14), and when TGF-β1 scavengers were used, the level of genomic damage in non-target cells was reduced (15), demonstrating the critical role of TGF-β1 in RIBE.

Radiotherapy can lead to DNA double-strand breaks in tumor cells, and RIBE can induce paracellular cells to undergo effects similar to it. Hu et al. demonstrated that TGF-β1 induces DNA damage in paracellular cells in their experiments (14), and in the previous study of our group, we have also demonstrated that RIBE can cause DNA double-strand breaks in neighboring lung mesenchymal stem cells through TGF-β1 (16). Homologous recombination repair (HR) is a key pathway for cells to repair DNA double-strand breaks and is essential for maintaining genomic stability. It has been reported in the literature that TGF-β1 can inhibit homologous recombination repair of DNA, and an abnormal increase in TGF-β1 can increase the phosphorylation of Samd3, whereas the TGF-β1/SMAD3 complex inhibits the formation of the BRCA1/RAD51 nuclear complex, a key protein for homologous recombination repair (8), which results in failure of DNA damage repair. However, how TGF-β1 regulates homologous recombination repair has not been clarified. Therefore, our study aimed to reveal the molecular mechanisms of how the TGF-β1/Smad3 signaling pathway inhibits DNA damage repair and promotes the paracrine effect of BMSCs after radiotherapy in lung cancer.

Recent studies have highlighted the primary cilium, a solitary, microtubule-based organelle protruding from the cell surface, as a key signaling hub that compartmentalizes various pathways, including TGF-β signaling. Specific receptors, including TGF-βRI, can localize to the ciliary membrane, facilitating efficient signal transduction. The assembly and maintenance of the primary cilium depend on intraflagellar transport (IFT), with IFT88 being a core component. It has been hypothesized that the cilium may orchestrate cellular responses to stressors like radiation, but its specific role in RIBE remains unexplored (17).

Given the protective effects of *Astragalus membranaceus* against radiation injury, we aimed to investigate whether the primary cilium/TGF-βRI/Smad3 axis is a central mechanism in RIBE affecting BMSCs and to identify specific *Astragalus* small molecules that could modulate this pathway to achieve both radioprotection of normal cells and radiosensitization of cancer cells.

## 2 Materials and methods

### 2.1 Bioinformatics analysis to find the target

Gene expression datasets were downloaded from the Gene Expression Omnibus (GEO) database. For radiosensitivity analysis, GSE20549 (6 resistant *vs*. 6 sensitive), GSE185698 (3 *vs*. 3), and GSE197236 (3 *vs*. 3) were utilized. For RIBE analysis, GSE8993 (4 control *vs*. 12 bystander), GSE18760 (4 *vs*. 4), and GSE21059 (4 *vs*. 4) were employed. Differential expression analysis was performed in R using the limma package with a stringent threshold of |logFC| >0.5 and *p*-value <0.05. The RobustRankAggreg (RRA) method was used to integrate ranked lists and identify robust intersection genes. The protein–protein interaction (PPI) network was constructed using the STRING database (confidence > 0.4) and visualized with Cytoscape (v3.8.1). The MCODE plugin was used to identify significant modules (settings: Degree Cutoff = 2, Node Score Cutoff = 0.2, K-Core = 2, Max. Depth = 100).

### 2.2 Cell culture

The BMSC line was from ScienCell Corporation (USA, Item No. 7500), and both the human lung adenocarcinoma cell line A549 and the human non-small cell lung cancer cell line H1299 were provided by the Provincial Key Laboratory of Research on Major Diseases Molecular Medicine and Prevention of Traditional Chinese Medicine in Universities and Colleges of Gansu Province. The A549 cells were cultured in F12 medium (Gibco), to which 10% bovine serum (Gibco (Thermo Fisher Scientific), Grand Island, New York, USA) and 1% penicillin–streptomycin mixture (Solarbio, Beijing, China) were added. Cells were cultured in 95% air and 5% CO_2_, and the temperature was kept at 37°C.

### 2.3 Co-culture model and irradiation

The irradiation was provided by the Heavy Ion Accelerator Cooled Storage Ring (HIRFL-CSR) at the Institute of Modern Physics, Chinese Academy of Sciences, with a dose rate of 2Gy/min (250 keV, 13.3 mA).

**Figure 1:**
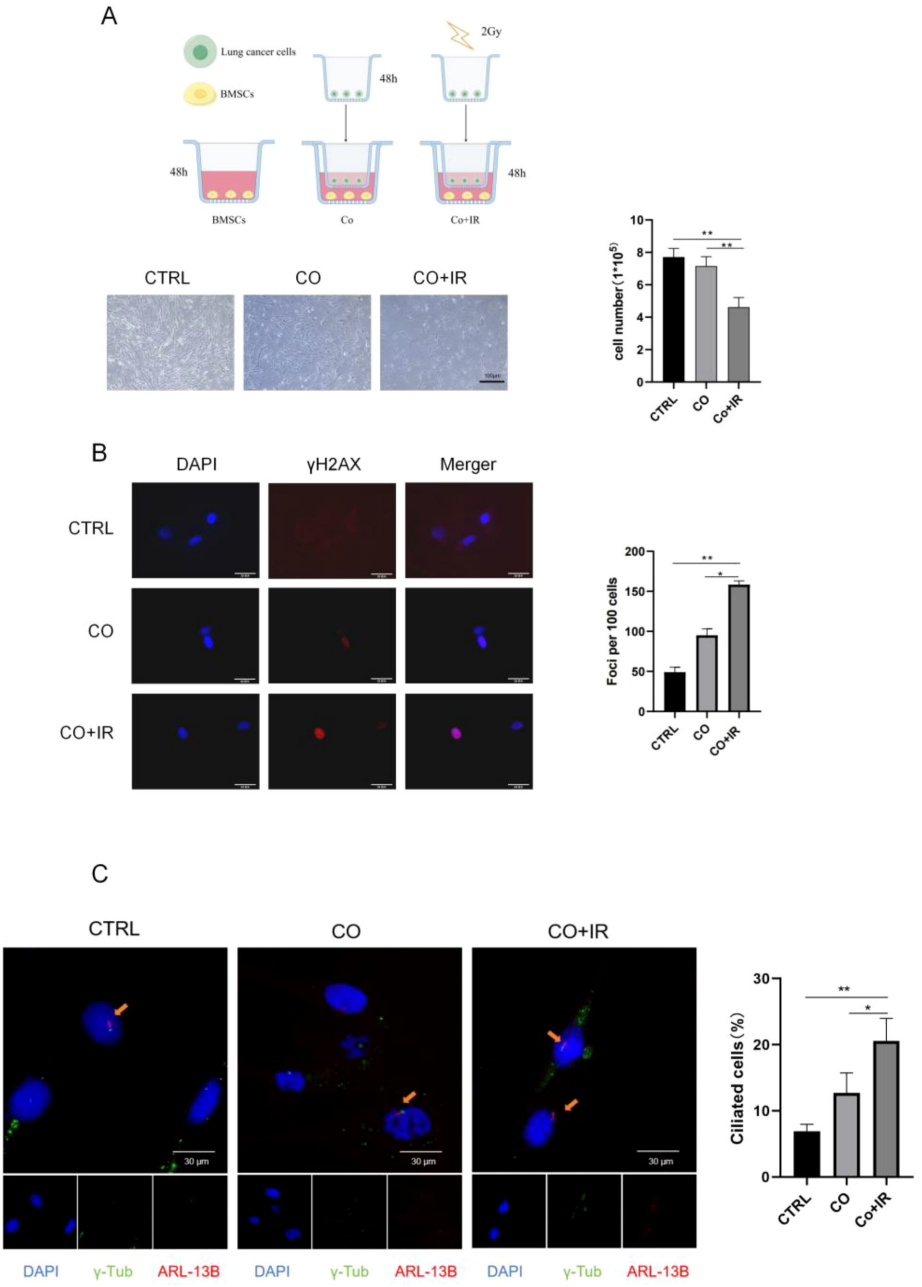
Primary cilia are key to DNA damage of adjacent BMSCs after irradiation. **(A)** Morphological photographs of cells in each group and a cell count plot of each group. **(B)** γ-H2AX fluorescence expression of paracellular stem cells 48 h after irradiation and γ-H2AX fluorescence foci plot of each group. **(C)** Paracellular cilium fluorescence expression 48 h after irradiation and ciliary growth rate analysis for each group. Data are presented as mean ± SD (*n* = 6). *p < 0.05, **p < 0.01.

A schematic of the co-culture model is provided in **Figure 1A**. A549 cells were seeded in the upper chamber of a Transwell insert at 2.4 × 10^4^ cells/mL, while BMSCs were seeded in the lower chamber at 5 × 10^4^ cells/mL. After adherence, only the upper chamber containing A549 cells was irradiated with 2 Gy of X-ray at a dose rate of 2 Gy/min. Control groups included a co-culture without irradiation (CO) and monocultures of either A549 or BMSCs with and without irradiation to distinguish direct effects from bystander effects.

### 2.4 Small interfering RNA transfection

IFT88 small interfering RNA (siRNA) (sequence: 5′-GCCGAAGTTCTTTACCAGATA-3′) and a scrambled siRNA (used as a negative control, NC) were transfected into BMSCs using Lipofectamine 3000 according to the manufacturer’s protocol. Transfection efficiency was confirmed by Western blot after 48–72 h.

Cells in the logarithmic growth phase were inoculated into 6-well culture plates at a density of 5 × 10^5^ cells per well, added with antibiotic-free medium, and cultured in an incubator containing 5% CO_2_ concentration for 24 h. Transfection was carried out when the degree of fusion reached 80%–90%. Instead of the culture medium, an Opti-MEM serum-free medium was used. Three microliters of transfection reagent was slowly added to 100 μL of Opti-MEM serum-free medium containing 1 μg of plasmid and incubated for 5–10 min at room temperature before being added dropwise to the 6-well plate and then gently mixed. After 24–72 h, the cells were observed under a microscope, and the transfection efficiency was detected by Western blot. The siRNA sequence was as follows (5′ to 3′): GCCGAAGTTCTTTACCAGATA.

### 2.5 Immunofluorescence staining

The 18-mm cell crawls were pre-inoculated into 12-well plates at a density of 5 × 10^4^ cells per well. Preparation of fluorescent samples involved paraformaldehyde fixation for 10 min, 0.1% Triton X-100 punching for 10 min, and blocking with 5% goat serum on a shaker for 2 h. Anti-Arl13b (17711-1-AP; Proteintech, Wuhan, China) and anti-β-tublin antibodies (ab137822; Abcam, Cambridge, United Kingdom) were used to detect primary cilia, and anti-γ-H2AX (ab26350; Abcam, Cambridge, United Kingdom) and anti-53BP1 antibodies (ab36823; Abcam, Cambridge, United Kingdom) were used to detect DNA damage. Images were captured under an RVL-100-G (ECHO) fluorescence microscope. Ten fields were randomly selected in at least three independent experiments, and the number of ciliated cells, total cell number, and the number of lesions were counted. Mean fluorescence intensity was analyzed using ImageJ software (Version 1.52; National Institutes of Health (NIH), Bethesda, Maryland, USA).

### 2.6 Western blotting

Total protein extraction of stem cells after co-culture radiation was performed, and 10% polyacrylamide gel was configured. An equal amount of samples per well was uploaded, and for the electrophoretic separation of protein, a stacking gel at 80 V for 30 min and a separating gel at 100 V for 90 min were used. The proteins were transferred to a PVDF membrane by the wet transfer method and blocked with 5% skimmed milk powder at room temperature for 2 h. Anti-53BP1 was incubated with the primary antibody (ab36823) or anti-GAPDH antibody (ab8245; Abcam, Cambridge, United Kingdom) overnight at 4°C or at room temperature for 3 h. The following day, the proteins were incubated with goat-anti-rabbit HRP-coupled secondary antibody (ab97051; Abcam, Cambridge, United Kingdom) or goat-anti-mouse HRP-coupled secondary antibody (ab97023; Abcam, Cambridge, United Kingdom) incubated at room temperature for 1.5 h. Imaging was developed with ECL luminol. The gray value was measured using ImageJ software, and the ratio of the gray value of the target protein bands of each group of samples to that of the internal reference bands was calculated as the relative expression level of the target protein. All antibodies used in this study are listed in **Supplementary Table 1**.

### 2.7 Flow cytometry detection of BMSC apoptosis rate

The BMSC cells of each group were made into a single-cell suspension, washed once with pre-cooled PBS, and centrifuged at 800 rpm for 5 min to collect the cell precipitate. A total of 300 μL of 1× binding buffer was added and the cells were resuspended by flicking and then filtered through a 300-mesh sieve into flow sampling tubes. Ten microliters of FITC-labeled Annexin-V solution and 5 μL of PI reagent were added to each tube and incubated for 5 min in the dark. A 200-μL 1× binding buffer was added, and the cells were sampled on a flow cytometer within 1 h for detection.

### 2.8 The CCK-8 method was used to detect the effect of irradiation on the proliferative ability of BMSCs

Cells in the logarithmic growth phase were collected and prepared as a single-cell suspension, inoculated into 96-well plates (5 × 10^3^ per well, 100 μL), and incubated at 37°C with 5% CO_2_ until the cells were attached to the wall (a cell counter was used to measure the concentration of the cells before spreading the plates). Ten microliters of CCK-8 solution was added at 12, 24, 48, 72, 96, and 120 h, and the plates were further incubated for 4 h before termination. The absorbance (OD) value of each group of cells was detected at 450 nm with a full-wavelength enzyme labeler.

### 2.9 Enzyme-linked immunosorbent assay

A diluent standard (1.0 mL) was added to the lyophilized standard and was allowed to stand for 15 min until it was fully dissolved and mixed, after which it was then diluted according to a certain concentration gradient. Then, it was sealed with a sealing film and was incubated for 30 min, washed five times with a washing solution and shaken dry, and 50 μL of enzyme reagent was added into each well, after which it was sealed again with a sealing film and was incubated at 37°C for 30 min. Each well was filled with a washing solution and left to stand for 30 s and then discarded, and this was repeated five times. Reagent A (50 μL) and reagent B (50 μL) were added to each well, shaken gently and mixed well, and color was developed for approximately 10 min at room temperature in the dark. A termination solution (50 μL) was added to each well to terminate the reaction. Zeroing was performed with a blank well, and the OD value was measured at 450 nm on an enzyme meter.

### 2.10 Statistical analysis

All data were analyzed using SPSS 23.0 software, and the results were expressed as mean ± standard deviation (± s). A *t*-test was used for two groups of data that conformed to normal distribution and for chi-square analysis, while one-way ANOVA was used for three or more groups of data. *p* < 0.05 indicated that the difference was statistically significant, and *p* < 0.05 indicated that the difference was significant.

## 3 Results

### 3.1 Radiation can cause DNA damage in adjacent BMSCs and increase the rate of primary cilium formation in BMSCs

A co-culture model of A549 cells and BMSCs was established using a Transwell system (**Figure 1A**). The results showed that radiation could induce injury to adjacent BMSCs. The number of BMSCs in the non-irradiated group was significantly lower than that in the irradiated A549 cells (*p* < 0.05) (**Figure 1A**). The expression of γ-H2AX was detected by immunofluorescence staining, and the DNA damage sites of BMSCs were significantly increased after co-culture. To more directly observe the number and status of cilia of adjacent BMSCs, we performed fluorescence staining experiments of cilia at 48 h of CO culture, and the results showed that the cilium-associated protein was elevated in the CO+IR group compared with the CO group, with abnormally increased cilia (*p* < 0.05) (**Figure 1C**).

### 3.2 The primary cilia/TGF-βR1 pathway plays a key role in the repair of radiation-induced DNA damage in adjacent BMSCs

To systematically investigate the common molecular basis of radiosensitivity and the RIBE in NSCLC, we first conducted a bioinformatics analysis. Gene expression datasets related to NSCLC radiosensitivity and RIBE were obtained from the GEO database.

**Figure 2:**
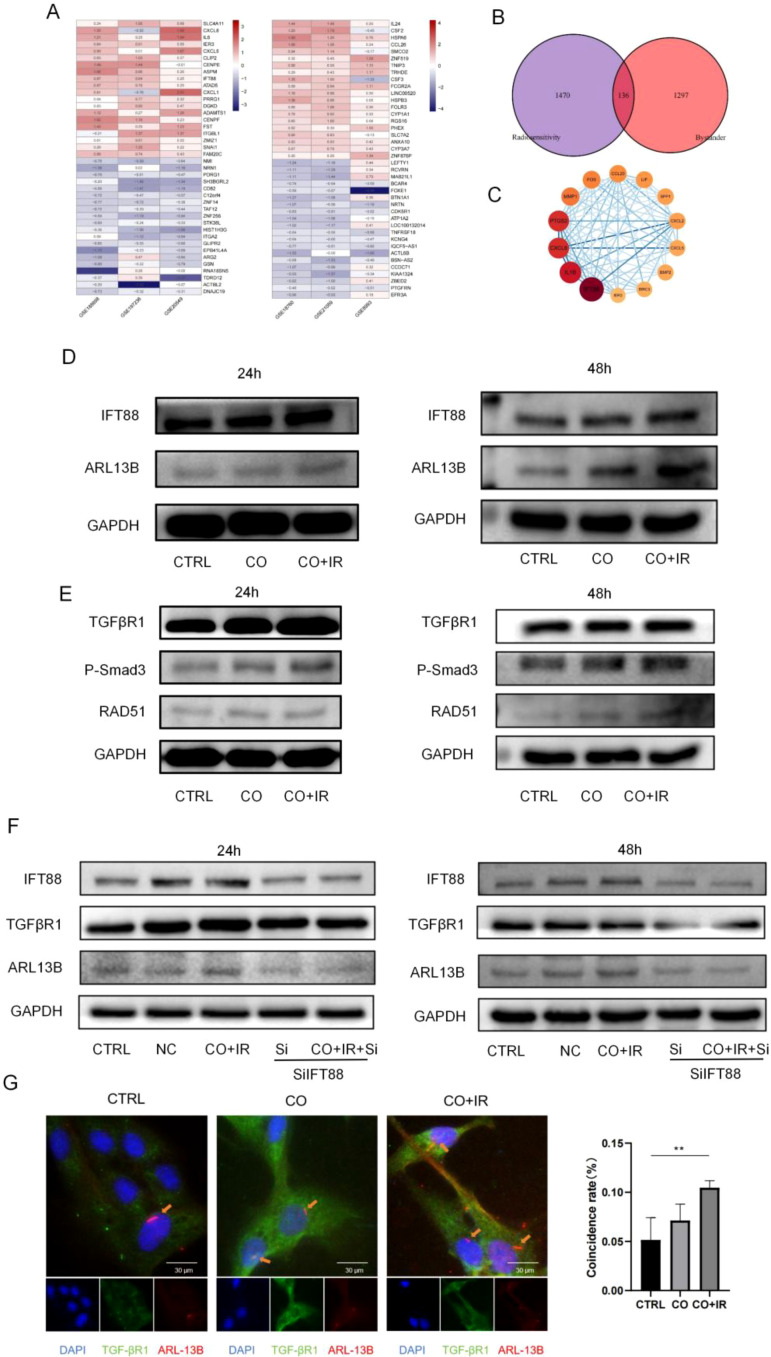
The primary cilia/TGF-βR1 pathway plays a key role in the repair of radiation-induced DNA damage in adjacent BMSCs. **(A)** To identify the intersection of the differential genes of the three datasets each for two radiation doses, RRA sequencing of the difference analysis results using the RobustRankAggreg package yielded the intersection difference genes of the three datasets; 1,606 and 1,433 differentially expressed genes were screened according to logFC absolute value greater than 0.5 and *p*-value less than 0.05. The heatmap package was used to display 20 up- and downregulated genes. **(B)** The differential genes for both were intersected and plotted in a Venn diagram, with 136 overlapping genes. **(C)** The core network consists of 14 genes; the darker the color, the greater the degree value and the more interactions in the protein network. The darker the color of the nodes, the higher the coexpression coefficient between the nodes. **(D)** Total cellular proteins of BMSCs were extracted at 24/48 h after radiation, and the expression levels of the ciliary-associated proteins ARL13B and IFT88 were verified by WB. **(E)** Total cellular proteins of BMSCs were extracted at 24/48 h after radiation, and the TGF-βR1 pathway-related protein expression was verified by WB. **(F)** After 2 Gy of X-ray radiation, A549 cells were co-cultured with BMSCs after Si intervention, and the total protein expression of ARL13B, IFT88, and TGF-βR1 in BMSCs was collected at 24/48 h **(G)** Analysis of cilia and TGF-βR1 fluorescence at 48 h after radiation and cilium coincidence with TGF-βR1 in each group. Data are expressed as mean ± standard deviation (*n* = 6). *p < 0.05, **p < 0.01.

For the radiosensitivity analysis, three datasets were included: GSE20549 (radioresistant *vs*. radiosensitive, *n* = 6 each), GSE185698 (*n* = 3 each), and GSE197236 (*n* = 3 each). Differential expression analysis was performed using the limma package (criteria: |logFC| > 0.5, *p* < 0.05), identifying 6231, 686, and 689 differentially expressed genes (DEGs), respectively (**Figure 2A**, the upper panel shows representative heatmaps and volcano plots, e.g., for GSE20549). To obtain robust common gene signatures, the RobustRankAggreg (RRA) method was employed to integrate and rank the results from the three datasets, yielding 1,606 intersection DEGs associated with radiosensitivity (**Figure 2A**).

For the RIBE analysis, three datasets were also included: GSE8993 (control *vs*. bystander, *n* = 4 *vs*. 12), GSE18760 (*n* = 4 *vs*. 4), and GSE21059 (*n* = 4 *vs*. 4). Differential analysis (same criteria) identified 717, 787, and 1,119 DEGs, respectively (**Figure 2A**). Integration via RRA yielded 1,433 intersection DEGs associated with RIBE (**Figure 2A**).

To further focus on key genes potentially involved in regulating both radiosensitivity and RIBE, the two sets of intersection DEGs (1,606 and 1,433) were overlapped, resulting in 136 common genes (**Figure 2B**). To decipher the PPI relationships among these 136 genes, they were imported into the STRING database to construct a PPI network (confidence > 0.4), which was visualized using Cytoscape. The preliminary network comprised 52 nodes and 163 interaction edges. Subsequently, network topology analysis was performed using the MCODE plugin to identify the most densely connected functional module. Parameters were set as follows: Degree Cutoff = 2, Node Score Cutoff = 0.2, K-Core = 2, and Max Depth = 100. This analysis identified a highly interconnected subnetwork consisting of 14 core genes (**Figure 2C**). This subnetwork contained 74 edges, suggesting close functional collaboration among these genes. The 14 core genes were as follows: *IEL3*, *BIRC3*, *BMP2*, *CXCL5*, *CXCL2*, *SPP1*, *LIF*, *CCL20*, *FOS*, *MMP1*, *PTGS2*, *CXCL8*, *IL1B*, and *IFT88*. Notably, *IFT88* encodes a key protein involved in intraflagellar transport and primary cilium formation. The specific fold changes (logFC) for these 14 core genes are provided in **Supplementary Table S1**.

Based on this analysis, we hypothesized that the primary cilium might play a significant role in RIBE signal transduction and cellular radiation response. To test this hypothesis and elucidate the function of IFT88, we proceeded with the following experiments.

The results showed that co-culture with irradiated A549 cells increased the expression of cilium and cilium-associated proteins, and the expression of IFT88 and ARL13B in the CO+IR group was significantly higher than that in the CO group at 24 and 48 h, and protein elevation was more pronounced at 48 h *(p* < 0.05) (**Figure 2D**; Ps1 A-B). The results showed that, compared with non-irradiation, the expression of TGF-βR1 and p-Smad3 in BMSCs increased at 24 and 48 h after irradiation, while the expression of RAD51 increased significantly at 48 h after irradiation (*p* < 0.05) (**Figure 2E**; Ps1C-D). Next, we knocked down IFT88, an siRNA knockdown of IFT88 (si-IFT88), for the elimination of primary cilia. Compared with the CO+IR group, the expression of TGF-βR1, p-Smad3, and RAD51 in the adjacent BMSCs was significantly decreased by Western blot (WB). Compared with the SI group, the expression of RAD51 in the CO+IR+Si group was significantly decreased (*p* < 0.05) (**Figure 2F**; Ps1E-F). To validate the critical role of primary cilia in the repair of DNA damage by radiation-induced paracentric BMSCs, colocalization of TGF-βR1 with Arl13b in paracentric BMSCs was observed using immunofluorescence staining after 48 h of co-culture (*p* < 0.05) (**Figure 2G**). Furthermore, qPCR analysis confirmed that radiation also upregulates IFT88 and ARL13B mRNA levels in BMSCs (**Figure Ps4**).

### 3.3 Paracellular apoptosis as well as primary cilia is reduced after small molecule intervention

At present, molecular docking has become an effective way for computer-aided screening of pharmacodynamic molecules. In order to find effective small molecules of *A. membranaceus*, the two targets TGF-βR1 and IFT88 were predicted by molecular docking and other network information technology. Previous studies by our research group have screened the small molecule vanillic acid for TGF-βR1 docking and determined the optimal concentration for the intervention of paracellular stem cells after irradiation (18). In order to screen the best small molecules to bind to IFT88, a series of explorations was carried out in this chapter. First, we predicted the protein structure of IFT88 by homology modeling, and then 87 kinds of small molecules of *A. membranaceus* were linked to IFT88. Some small molecules with high scores were screened out, and these were isorhamnetin, rhamnoside, *Alpinia officinalis*, isorhamnetin, kaempferol, coumarin, calycosin isoflavone, larch resinol, 3-hydroxy-9,10-dimethoxy rosewood, trans-isoferulic acid, and vanillic acid. Combined with the rest of the pre-group screen for *Astragalus* small molecules for stem cells, six kinds of ideal small molecules were selected, which were vanillic acid (VA), isoglycyrrhizin (II), onosanthin (On), daidzein (Da), isosanthin (Im), and rosewood (Me). The molecular docking binding energies of IFT88 with the six small molecules are presented in **Supplementary Table S3**.

**Figure 3:**
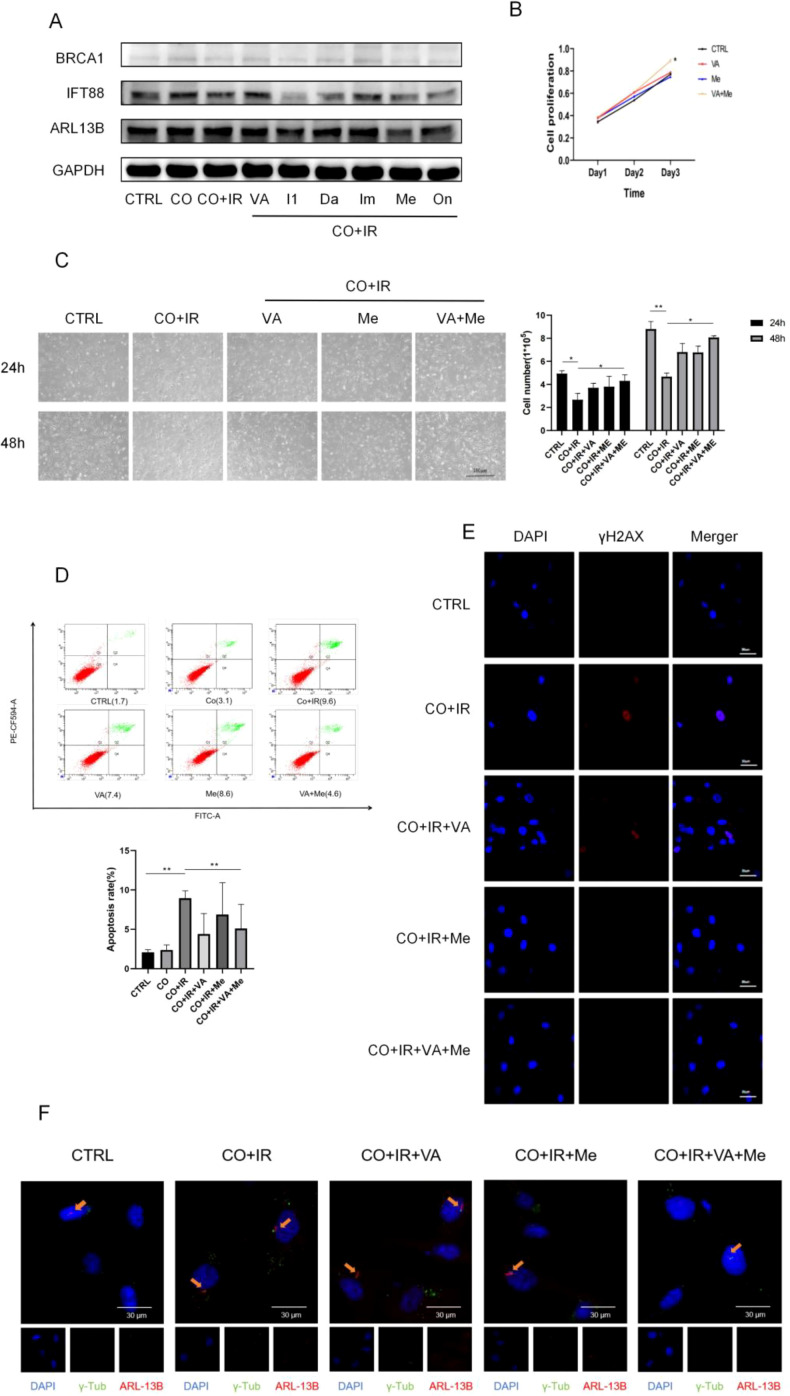
Molecular docking screening for the best *Astragalus* small molecules, apoptosis, and primary cilia after small molecule intervention. **(A)** After 2 Gy of X-ray radiation, A549 cells were co-cultured with BMSCs, and the total protein expression of ARL13B, IFT88, and BRCA1 in BMSCs was extracted for 48 h **(B)** CCK-8 for the optimal concentration of small molecules for the proliferation of BMSCs after radiation. **(C)** Morphological photographs of cells of each group and statistics of the cell count of each group. **(D)** Analysis of apoptosis by each group. **(E)** γ-H2AX at 48 h after radiation. **(F)** 48 h after radiation. Data are expressed as mean ± standard deviation (*n* = 6). *p < 0.05,**p < 0.01 < 0.01.

Using these six small molecules to interfere with paracellular stem cells, WB detected the primary cilium-associated proteins, and the results showed that the protein expression levels of IFT88 and ARL13B in paracellular stem cells after irradiation could be significantly reduced by zetalane (*p* < 0.05) (**Figure 3A**; Ps 1G); therefore, this subject selected rosewood and vanillic acid as IFT88 and TGF-βR1 double targets of small molecules for follow-up experiments (**Figure 3B**).

In conclusion, we selected vanillic acid (25 μmol/L) and 3-hydroxy-9,10-dimethoxy rosewood (6.25 μmol/L) from *A. membranaceus* small molecules as drugs for the treatment of radiation side effects and carried out follow-up experiments.

Based on the above-screened small molecules targeting the two key targets, we next explored the effect of small molecules alone or in combination on the paradiotropic effect of BMSCs after radiotherapy for lung cancer. It was found that the two small molecules could effectively improve the cell state of irradiated BMSCs (**Figure 3C**) and reduce the apoptosis and DNA damage of BMSCs (**Figures 3D, E**; Ps 1H). Then, we used WB experiment and immunofluorescence experiment to find the small molecules that can reduce the effect of primary cilia, especially after superposition of the drugs, and compared with the single use of small molecules, the number of primary cilia in stem cells was significantly reduced after irradiation (**Figure 3F**; Ps1 I).

### 3.4 Small molecules act through the primary cilia as well as through TGF-βR1

**Figure 4:**
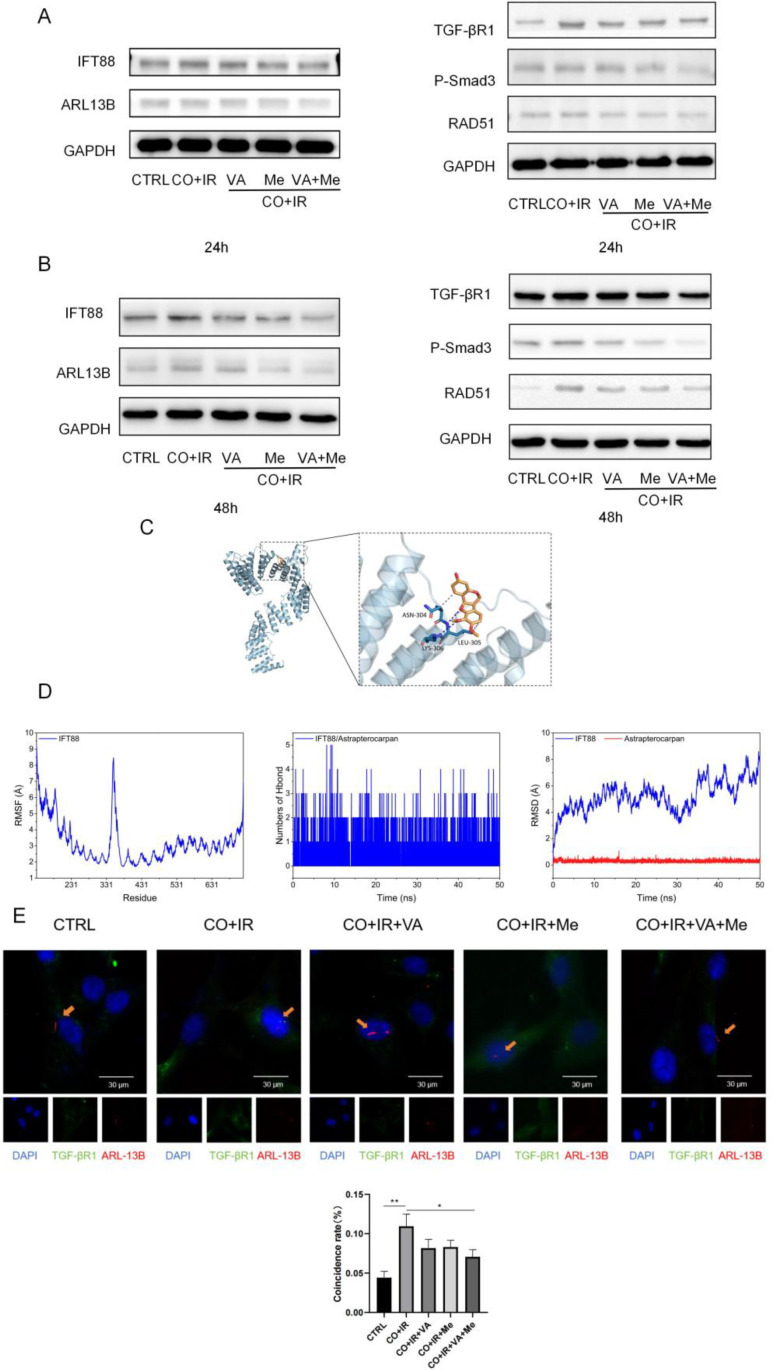
Small molecules act through the primary cilia as well as TGF-βR1. **(A)** After 2 Gy of X-ray radiation, A549 cells were co-cultured with BMSCs, total cellular proteins of BMSCs were extracted 24 h after drug intervention, and protein expression of cilia/TGF-βR1-related cilia was verified by WB. **(B)** After 2 Gy of X-ray radiation, A549 cells were co-cultured with BMSCs, the total cellular protein of BMSCs was extracted for 48 h after drug intervention, and the protein expression of cilia/TGF-βR1 was verified by WB. **(C)** Docking diagram of small molecules and IFT88 molecules. **(D)** Calculation of the root mean square fluctuation (RMSF) based on the molecular dynamics simulation trajectory, and the number of hydrogen bonds between small molecules and proteins changes during the molecular dynamics simulation. Complex root mean square bias (RMSD) difference over time during molecular dynamics simulation. Data are expressed as mean ± standard deviation (*n* = 6). *p < 0.05, **p < 0.01.

Based on the above-selected small molecules for two key targets, we used WB experiments to find the small molecules that can reduce related proteins of the primary cilium/TGF-βR1 after 24/48 h. Especially after superimposed administration, the primary cilium protein/TGF-βR1 of radiation stem cells was significantly reduced compared with the small molecule alone (**Figures 4A, B**; Ps 2A-B). We found that compared with no drug, both small molecules, either alone or superimposed, inhibited the TGF-βR1 pathway-related protein and the DNA damage repair protein RAD51, with the best effect. In order to determine the connection between the selected small molecules and the selected key targets, we also conducted molecular dynamics simulation experiments, in which we found that vanilloid acid and TGF-βR1 and IFT88 have a stable binding relationship (**Figures 4C, D**). Then, in order to verify the mechanism of action of the small molecules, we found that the coincidence rate of TGF-βR1 and Arl13b proteins was significantly decreased in the drug-added group, which was consistent with previous experiments (**Figure 4E**).

### 3.5 Small molecules do not act through TGF-β1, and small molecules have the effect of increasing the sensitivity of radiotherapy to cancer cells

In order to determine whether the Pscs were affected by TGF-β1, we examined the TGF-β1 content in the stem cell culture media of each group after radiation dosing. The results showed that radiation could cause the increase in TGF-β1, whereas there was no significant decrease after dosing (Ps 3A).

**Figure 5:**
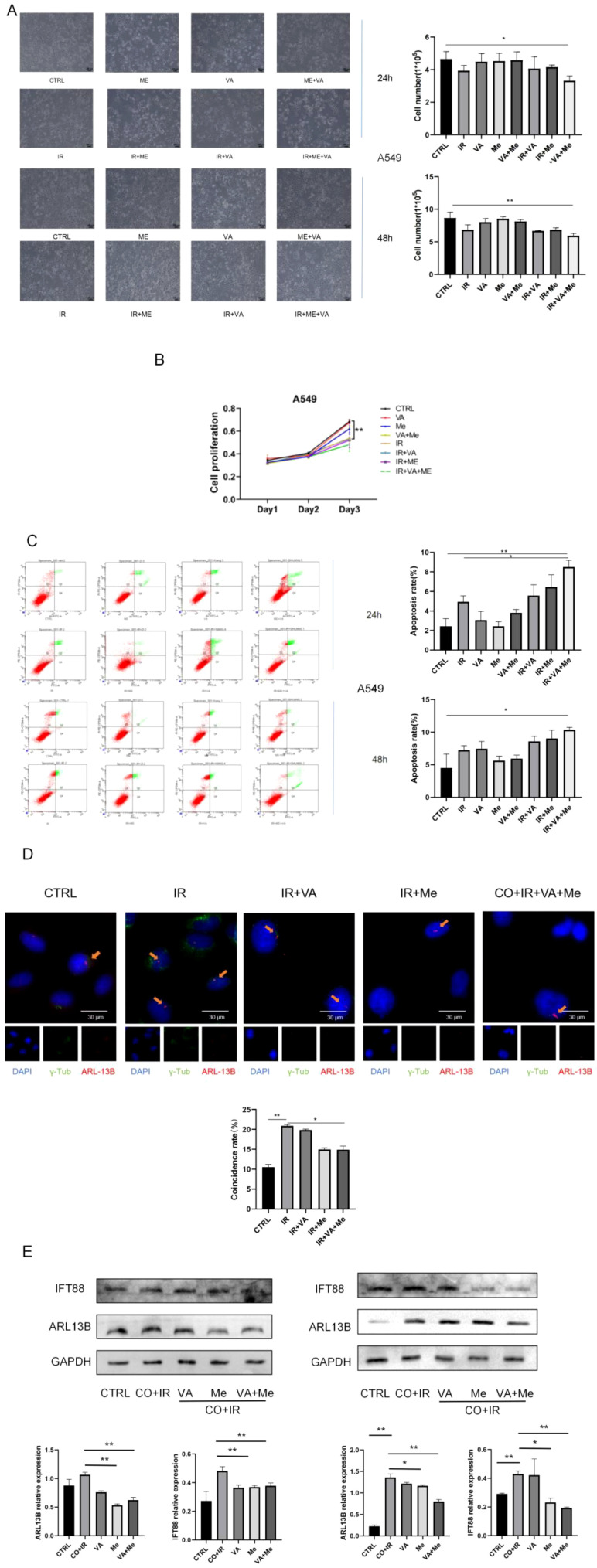
A549 and H1299 cells after *Astragalus* small molecules and compatibility intervention with 2 Gy of radiation. **(A)** Morphological photographs of A549 cells 24/48 h and the statistical map of cell counts in each group. **(B)** A549 proliferation. **(C)** Post-radiation of lung cancer, A549 expression of cell apoptosis at 24/48 h **(D)** Cell cilia fluorescence expression 48 h after radiation and cilia growth rate analysis of each group. **(E)** A549 cells 48 h after intervention; WB verified the expression of the cilia-related proteins ARL13B and IFT88. Data are expressed as mean ± standard deviation (*n* = 6). *p < 0.05, **p < 0.01.

To verify whether these two small molecules also play a role in enhancing the radiosensitivity of tumor cells, we selected two lung cancer cell lines—A549 and H1299—which were subjected to the same radiation (2 Gy) as the paracellular model with drug intervention. In this part, we observed cell morphology and used a flow cytometry CCK-8 experiment to detect apoptosis and proliferation (**Figures 5A–E**; Ps2 C-D), and the results showed that neither the single drug nor the superimposed drug had a killing effect on lung cancer cells, although the killing effect on tumor cells was stronger after the superimposed drug was given.

## 4 Discussion

Radiotherapy, a mainstay in lung cancer treatment, is paradoxically limited by two major phenomena: the collateral damage inflicted upon normal tissues via the RIBE and the intrinsic or acquired radioresistance of cancer cells. This study provides novel insights into both challenges (5, 19). We identify the primary cilium as a critical signaling hub that transduces RIBE signals in bystander BMSCs via the TGF-βR1/Smad3 pathway, thereby suppressing DNA repair and exacerbating genomic injury. Furthermore, we demonstrate that two natural compounds derived from *A. membranaceus*—VA and 3-hydroxy-9,10-dimethoxypterocarpin (Me)—can simultaneously protect BMSCs from RIBE and enhance the radiosensitivity of lung cancer cells, unveiling a promising dual-target and dual-effect strategy to improve therapeutic outcomes.

Our bioinformatic analysis, integrating datasets from both radiosensitive and RIBE models, pinpointed IFT88 as a core overlapping gene. IFT88 is an essential component of the intraflagellar transport machinery, required for the assembly and maintenance of the primary cilia. This finding led us to hypothesize a functional link between ciliogenesis and the cellular response to radiation stress. Using a physiologically relevant co-culture model, we confirmed that RIBE not only induces DNA damage (γ-H2AX foci) in bystander BMSCs but also triggers a significant increase in primary cilium formation. This is consistent with emerging evidence that the cilium acts as a central sensor for extracellular stressors (17). More importantly, we established a causal relationship: siRNA-mediated knockdown of IFT88, which disrupts ciliogenesis, profoundly attenuated the RIBE-induced upregulation of the TGF-βR1/Smad3 pathway and its downstream effector RAD51. This compellingly demonstrates that an intact primary cilium is necessary for the full activation of the TGF-βR1-mediated DNA damage response in the bystander context. Our data support a model wherein RIBE signals (potentially including TGF-β1) promote ciliary assembly, which in turn facilitates the concentration and activation of TGF-βR1 within this specialized compartment, leading to Smad3 phosphorylation and subsequent inhibition of homologous recombination repair.

To translate this mechanistic understanding into a therapeutic strategy, we employed a structure-based approach to screen for natural modulators. We selected VA for its known interaction with TGF-βR1 and identified Me as a novel predicted binder of IFT88 through molecular docking. The efficacy of this dual-target approach was remarkable. Both compounds, especially in combination, reduced ciliogenesis, mitigated DNA damage, and decreased apoptosis in BMSCs subjected to RIBE. A key mechanistic detail, responsive to the reviewer’s insight, is that while total TGF-βR1 protein levels remained unchanged, VA+Me treatment significantly reduced the colocalization of TGF-βR1 with the ciliary marker Arl13b. This suggests that these small molecules exert their effect not by downregulating the receptor but likely by impairing its proper trafficking to or stabilization within the ciliary membrane, possibly through modulating IFT88 function or ciliary architecture, thereby blunting signal transduction.

The most striking and clinically relevant finding is the differential effect of VA and Me on normal versus cancer cells. While protecting stromal BMSCs, the same compounds sensitized A549 and H1299 lung cancer cells to radiation. This radiosensitization was associated with a reduction in ciliary proteins in the cancer cells. We speculate that this dichotomy may arise from the distinct biological roles and dependencies of the primary cilium in different cell types. In stem-like BMSCs, the cilium may be crucial for coordinating pro-survival and repair pathways in response to microenvironmental stress. Its disruption by our compounds might therefore abrogate a key adaptive mechanism, rendering the cells more susceptible to RIBE. In contrast, in rapidly dividing cancer cells, which often downregulate cilia, residual ciliary signaling might contribute to stress resistance or DNA repair. Disrupting this vestigial pathway with VA and Me could then selectively enhance radiation-induced killing. This “cilium-dependent synthetic lethality” hypothesis warrants further investigation but highlights the therapeutic potential of targeting organelle-specific signaling.

Our study has limitations. The *in vitro* co-culture model, although controlled, cannot capture the full complexity of the tumor microenvironment *in vivo*. Future validation in animal models is essential. Additionally, the precise binding mode of Me to IFT88 and its exact effect on IFT complex function require further biochemical and structural elucidation.

In conclusion, this work uncovers the primary cilium/TGF-βR1 axis as a pivotal mechanism driving RIBE-induced damage in lung stromal cells. By rationally selecting two *Astragalus*-derived small molecules to target this pathway at two nodes (IFT88 and TGF-βR1), we achieved a dual beneficial outcome: radioprotection of normal BMSCs and radiosensitization of tumor cells. This approach moves beyond traditional single-target strategies and aligns with the holistic, multitarget philosophy of traditional medicine. It presents an innovative paradigm for developing adjuvants that widen the therapeutic window of radiotherapy, making it both safer and more effective.

## Data Availability

The datasets presented in this study can be found in online repositories. The names of the repository/repositories and accession number(s) can be found below: https://www.ncbi.nlm.nih.gov/geo/, GSE20549\GSE185698\GSE197236\GSE21059GSE8993\GSE18760\.
