# Supplementary material for: Astragalus small molecules protect BMSCs from radiation-induced bystander effect and enhance lung cancer radiosensitivity via the primary cilium/TGF-βR1/Smad3 pathway
Source: Front Oncol. 2026 Mar 4;16:1732029. doi: 10.3389/fonc.2026.1732029 (PMC12995683; doi:10.3389/fonc.2026.1732029)
Supplement: Supplementary file 1 [file DataSheet1.docx]

### ****Supplementary Table 1.****

| Target | Host | Vendor | Catalog # | Application (Dilution) |
| --- | --- | --- | --- | --- |
| IFT88 | Rabbit | Proteintech | 13967-1-AP | WB (1:1000) |
| ARL13B | Rabbit | Proteintech | 17711-1-AP | IF (1:200), WB (1:1000) |
| TGFβRI | Rabbit | Cell Signaling | 3709S | WB (1:1000), IF (1:200) |
| p-Smad3 | Rabbit | Cell Signaling | 9520S | WB (1:1000) |
| RAD51 | Rabbit | Abcam | ab133534 | WB (1:2000) |
| γ-H2AX | Mouse | Millipore | 05-636 | IF (1:500) |
| GAPDH | Mouse | Proteintech | 60004-1-Ig | WB (1:5000) |

All antibodies used

**Supplementary Table2**

| GSE20549 , GSE185698 , GSE197236 | | |
| --- | --- | --- |
| Name | pvalue | logFC |
| IFT88 | 0.00003382617 | 0.852821664 |
| IL1B | 0.007087233 | 0.689200019 |
| CXCL8 | 0.0000068 | 1.966200391 |
| PTGS2 | 0.000351852 | 1.088011937 |
| MMP1 | 0.003151122 | 1.431398775 |
| FOS | 0.000209284 | 0.961818928 |
| CCL20 | 0.004060774 | 0.96301726 |
| LIF | 0.000967894 | 1.517356042 |
| SPP1 | 0.012240429 | 0.944458657 |
| CXCL2 | 0.000221796 | 1.24095505 |
| CXCL5 | 0.0000220980168072895 | 1.908751237 |
| BMP2 | 0.006978287 | -0.559984153 |
| BIRC3 | 0.012319147 | -0.989167635 |
| IER3 | 0.000019179881572537 | 1.120075107 |

| GSE8993, GSE18760, GSE21059 | | |
| --- | --- | --- |
| Name | pvalue | logFC |
| IFT88 | 0.002402725 | 0.950391779 |
| IL1B | 0.005608591 | 0.923715647 |
| CXCL8 | 0.001889274 | 1.770917814 |
| PTGS2 | 0.019147882 | 1.288678315 |
| MMP1 | 0.003227567 | 1.429367837 |
| FOS | 0.007578256 | 0.832802392 |
| CCL20 | 0.001364739 | 1.961386363 |
| LIF | 0.016329767 | 0.764977358 |
| SPP1 | 0.009132711 | 1.881706706 |
| CXCL2 | 0.00702699 | 1.172985925 |
| CXCL5 | 0.010818981 | 1.119065736 |
| BMP2 | 0.022708717 | 0.807063085 |
| BIRC3 | 0.014536361 | 1.238557798 |
| MIER3 | 0.036389676 | 0.850004232 |

The logFC values for the core 14 genes are now provided in the main text

**Supplementary Table3**

IFT88:

| NO. | Name | Docking score |
| --- | --- | --- |
| 1 | Me | -5.32 |
| 2 | VA | -4.80 |
| 3 | II | -3.37 |
| 4 | On | -4.98 |
| 5 | Da | -5.21 |
| 6 | Im | -5.23 |

TGFBR1:

| NO. | Name | Docking score |
| --- | --- | --- |
| 1 | Me | -6.00 |
| 2 | VA | -6.06 |
| 3 | II | -6.85 |
| 4 | On | -6.38 |
| 5 | Da | -7.30 |
| 6 | Im | -6.51 |

The molecular docking binding energies of IFT88 with the six small molecules
